# Polypyrrole-wool composite with electrical heating properties fabricated via layer-by-layer method

**DOI:** 10.1038/s41598-024-54678-6

**Published:** 2024-02-16

**Authors:** Suhyun Lee

**Affiliations:** Department of Fashion and Textiles, Seoul National University, Seoul, 08826 South Korea

**Keywords:** E-textiles, Electrical heating performance, Polypyrrole, Wool felt, Layer-by-layer, Conductive fabrics, Nanoscale materials, Soft materials, Organic chemistry

## Abstract

This study presents the development of conductive polymer-textile composites with outstanding electric heating properties achieved through the in-situ polymerization of polypyrrole on wool-felt fabrics, renowned for their superior thermal insulation. Employing successive layer-by-layer (LBL) cycles facilitated precise control over the uniform deposition of polypyrrole with the fabrics. The investigation focused on the interaction between wool fiber and polypyrrole, evaluating appearance, add-on, and electrical heating performance with varying LBL cycles. The polymerization process resulted in the formation of spherical polypyrrole particles on the wool-felt, with deposition increasing alongside LBL cycle numbers. Mechanical properties, including tensile strength and bending rigidity, exhibited enhancement with polypyrrole deposition, while strain reduction was noted, with minimal influence from LBL cycles. Electrical properties, particularly surface resistance, displayed a rapid decrease up to the second LBL cycle. Concerning electrical heating performance, the application of a 12 V voltage resulted in a linear increase in surface temperature with increasing LBL cycles, peaking at 15.5 ℃. Notably, this sustained electrical heating effect persisted even after voltage removal, attributed to the low thermal conductivity of wool fiber. Moreover, the polypyrrole conductive layer maintained exceptional conductivity following repeated abrasion and washing, credited to improved uniformity through LBL cycles. The synergy of wool's insulating properties and polypyrrole's conductivity, as confirmed in this study, presents the potential for a highly efficient heating fabric. These developed materials exhibited improved heating performance, energy conservation, and minimal change in mechanical properties, making them suitable for applications such as electrical heating smart clothing.

## Introduction

Textile composites with electrical conductivity properties, called e-textiles, are drawing interest from both the industry and the academia because of their suitability for wearable devices and smart clothing in applications such as sensing, energy harvesting, heating, and location trackings^1–4^. Electronic textiles with high dimensional flexibility cannot be fabricated using conventional electronic substrates. The processing of e-textiles involves the convergence of textiles and electronics to develop smart materials with various functions, typically found in inflexible and rigid devices^5^. In previous studies, conductive materials, such as metal nanoparticles and nanowires, carbon nanotubes, graphene, graphite, and conductive polymers have been doped into or deposited on fabric to prepare e-textiles^2,6^.

Conductive polymers, including polyaniline, polythiophene, and polypyrrole, have attracted the attention of researchers because they are lightweight, environmentally stable, compatible with insulator materials, and their tunable conductivity can be tuned by changing their fabrication conditions^1,3,7^. In particular, because tuning the electrical resistance of these materials can generate an electrical response when the fabric surface is exposed to external stimuli, these polymers can be modified with the desired properties by incorporating various nanomaterials into the matrix^8^. Thus, conductive polymer-coated fabrics can be fabricated to possess desirable combinations of properties, such as high conductivity, flexibility, strength, and structural variability^9^. In particular, fabrics combined with conductive polymers are used as electrothermal materials because they can convert electrical energy into heat without requiring electrical wiring^10,11^.

Polypyrrole, as a conductive polymer, offers advantages such as nontoxicity, easy polymerization, high conductivity, and excellent atmospheric and thermal stability compared to other conductive polymers^11,13–15^. However, these polymers are insoluble and do not melt due to strong interaction within the polymer chain, making their application to fabrics challenging using standard coating method^16^. Generally, polypyrrole can be polymerized and integrated into fabrics through chemical or electrochemical methods, such as in-situ polymerization, emulsion polymerization, and vapor-phase polymerization^17,18^.

The adhesion of conductive polymers to fabrics is typically low, posing a significant challenge to the commercial application of conductive polymer-coated fabrics. The primary obstacle lies in the poor adhesion of the coating to the fiber surface^8,19^. Therefore, enhancing adhesion strength between conductive polymers and fabrics is crucial, involving both chemical bonds and physical interactions. Among various fabrication methods, the layer-by-layer (LBL) approach has been employed to achieve the uniform preparation of conductive materials^16,20^. He et al.^21^ introduced a novel and straightforward method for preparing conductive fabrics with polyaniline (PANI) using LBL assembly technology, utilizing nylon as the substrate. They confirmed that the conductivity of the composite fabric increased with the number of assembly times, indicating enhanced absorption of PANI by the nylon.

Among the various fibers, wool is an important source of natural fibers^13^. Wool has low thermal conductivity and is suitable for keeping warm in cold weather, so it is used for winter clothing and insulation^22^. Therefore, by utilizing the low thermal conductivity of wool fibers, it is anticipated that introducing heating performance of conductive materials can achieve high temperatures and excellent insulation durability with minimal energy consumption. Wool is primarily composed of the protein α-keratin and a crystalline polypeptide with contiguous polypeptide chains crosslinked by disulfide bonds from the amino acid cystine^23^. This crosslinking renders keratin relatively hard and insoluble, making it difficult for pyrrole monomers to enter the fibers for polymerization^13^. However, Chatterjee and Maity^24^ identified wool as the most effective substrate for in-situ polymerization of pyrrole. Coating various fibers, they found wool exhibited the highest polypyrrole add-on due to its scales and irregular surfaces, providing superior anchoring points and potential chemical interactions. Therefore, if a composite is made using wool fibers and polypyrrole, it can be used as an e-textile with excellent electrical heating performance, flexibility, and light weight. In particular, the direct polymerization of polypyrrole on wool fabrics is a simple process that can increase their durability by improving the adhesion between the conductive polymers and fabrics^14^. Nonetheless, the hydrophobicity of the cuticles on the surface of wool fiber may impede the initial absorption and deposition of polypyrrole^25^. Thus, there is a need for the formation of a uniform polypyrrole layer through the introduction of a repeated polymerization process such as LBL method.

This study aimed to develop a conductive fabric with electrical heating properties through the in-situ polymerization of a polypyrrole onto fabrics. Wool felt was employed as a substrate to increase the bonding force with polypyrrole. Wool felt combines a three-dimensional structure with and overhead network and an open-channel architecture, rendering it an ideal substrate for e-textiles with polypyrrole, especially smart heating clothing^13^. The conductive polymer was deposited directly onto the wool felt fabric surface using an LBL method, that made it possible to uniformly deposit large quantities of polypyrrole on the wool felt^26^. To evaluate the effect of the polypyrrole deposition process on the wool fabrics, the changes in the appearance, mechanical, and chemical properties according to the number of LBL cycles were confirmed. The conductive properties of the fabricated polypyrrole-wool composites were analyzed based on their surface resistance, electric heating performances, and changes in surface resistance with abrasion and washing.

## Experimental section

### Materials

Wool felt fabrics (100%) were used as substrates. The weight and thickness of the wool felt were 223 g/m^2^ and 1.0 mm, respectively. The monomer selected for the in-situ polymerization of the conductive polymer was pyrrole (99% purity, Sigma-Aldrich, USA). Iron (III) chloride hexahydrate (Kanto Chemical Co., Inc., Japan) was used as the oxidants. All samples were used as-is without further purification.

### Preparation of polypyrrole-wool composite

**Figure 1:**
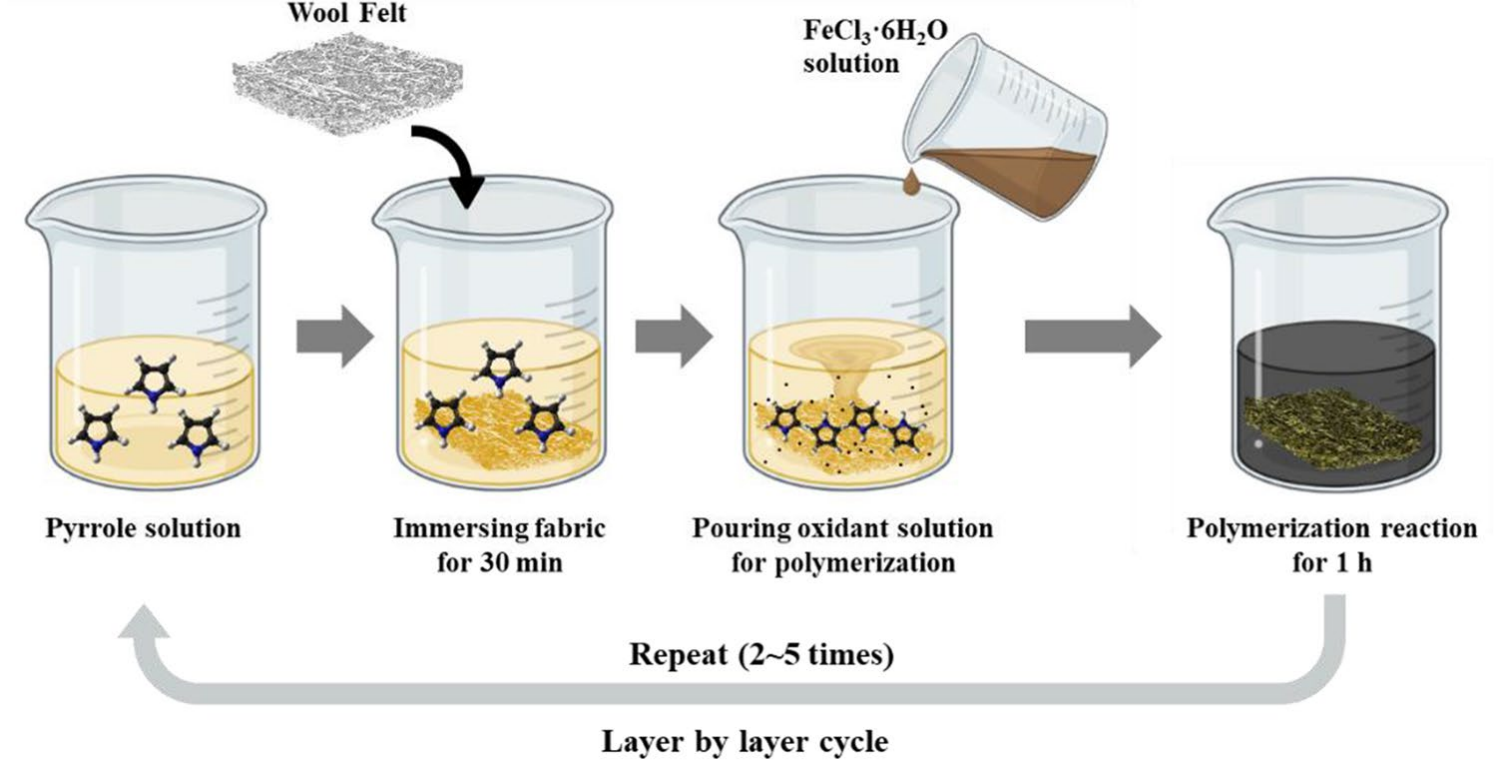
LBL process for polypyrrole-wool composites preparation.

The in-situ polymerization method proposed by Huang et al.^27^ was used for the preparation of polypyrrol-wool composites. As a monomer, 0.1 mol pyrrole was dissolved in distilled water (100 mL). After cutting the wool felt fabric into 10 × 10 cm squares, the pieces were immersed in the pyrrole solution and maintained at room temperature for 30 min so that the pyrrole monomers could be sufficiently absorbed by the fabrics. Subsequently, 0.2 mol of iron(III) chloride hexahydrate (FeCl_3_∙H_2_O) was dissolved in 50 mL of distilled water to create an oxidant solution which was poured into the pyrrole monomer solution where the wool fabric was immersed to initiate polymerization. The polymerization reactions of the polypyrrole deposition were performed for 1 h. After the completion of the reaction, the sample was removed and washed with distilled water. This procedure was considered as one LBL cycle and was repeated two, three, four, and five times. After the LBL processing, the samples were dried at room temperature. A schematic of the polymerization process is shown in Fig. 1.

### Characterization

#### Appearances

The surface morphologies of the polypyrrole-wool composites were observed using field-emission scanning electron microscopy (FE-SEM, Gemini500, Carl Zeiss, Germany) according to the number of LBL cycles. To prevent the samples from charging, their surfaces were coated with platinum using a sputter coater (108auto, Cressington Scientific Instrument, Ltd., UK) at a current of 30 mA for 120 s.

The add-on (%) of the fabrics due to the deposition of polypyrrole was calculated using Eq. (1):

1 $$ {\text{Add}} - {\text{on}}\;(\% ) = \left( {\frac{{W_{a} - W_{b} }}{{W_{b} }}} \right) \times 100 $$

where W_a_ is the weight of the samples after the LBL process of polypyrrole deposition and W_b_ is the weight of the samples before.

#### Chemical compositions and mechanical properties

The chemical structures of the polypyrrole and wool fabric before and after the deposition of polypyrrole were identified using an energy-dispersive X-ray spectrometer (EDS, X-Max Extreme, Oxford Instruments, UK) and a Fourier-transform infrared (FT-IR) spectrometer (Vertex80v, Bruker, USA). In the case of polypyrrole, it was polymerized alone, filtered, and dried at 100 ℃ to obtain polypyrrole powder for FT-IR analysis. The samples were scanned in the wavenumber range from 4000 to 600 cm^−1^ with a resolution of 8 cm^−1^.

The changes in the mechanical properties of the polypyrrole-wool composites were analyzed based on their tensile strength and stiffness. The tensile strength was measured by the ASTM D 5035 strip method utilizing a universal testing machine (Instron-5543, USA) with a 1 kN load at an extension rate of 30 cm/min. Stiffness was assessed using the ISO 4606: 2013 measurement method with a fixed-angle bending tester.

#### Conductivity

The surface resistivities of the polypyrrole-wool composites were measured using a DC milliohm meter (GOM-804, GW INSTEK, Taiwan) in accordance with the AATCC 76-1995 standard.

The electric heating properties were observed in relation to the surface temperature by applying a voltage using a thermal imaging camera (Testo 868, Testo SE&Co., Germany), to investigate the heat resistance of the polyprrole-wool composites. Voltages of 3, 6, 9, and 12 V were applied to the samples using a DC power supply (TS3020A, Toyotech, Japan). Thermal images of the surface were captured 1 min after applying voltage. The average surface temperature was calculated by extracting the area corresponding to the fabric sample from the captured image using the IRSofte software (Testo, Germany).

To evaluate the thermal properties of the clothing after the electric heating of the polypyrrole-wool composites, an evaluation model modified from the ISO 11,092 standard was created. The hot plate was maintained at 35 ℃ to simulate human skin conditions, and a still air layer was formed by leaving a gap of 1 cm between the sample and the hot plate. The sample was placed on a hot plate, and the temperatures of the still air layer and the sample surface were measured at 30 s intervals using a thermo recorder (TR 72A, T&D Corporation, Japan) and a thermal imaging camera, respectively. For the polypyrrole-wool composites, a voltage of 9 V was applied using a DC power supply, and the electricity supply was stopped 10 min after the start of the experiment. The evaluation was conducted for a total of 15 min in the case of no sample, the raw wool felt, and the L4 of polypyrrole-wool composite. The environmental conditions for this experiment were 22 ± 0.1 ℃ and 50 ± 3% RH.

#### Durability

The physical and chemical durability of the polypyrrole layer according to the number of LBL cycles was evaluated via an abrasion and washing test.

The abrasion test was conducted using a grinder (GEX 125-1 A/AE, Bosch, Germany). The sandpaper was p400 with a diameter of 125 mm and was rotated clockwise in contact with the sample surface under the speed of 7500 rpm. The rotation was repeated 60 times, and the surface resistance was measured every ten times. The surface resistance of the sample after repeated abrasion tests was obtained by performing the experiment in triplicate and calculating the average values.

For the washing durability test, the polypyrrole-wool composite samples were laundered using a front-loading drum washing machine (WF21B6400KP, Samsung Electronics, Republic of Korea) with a wool cycle. This wool cycle, integrated into the washing machine, operates at a washing temperature of 40 ℃, with 3 rinsing cycles, a spindle speed set to 2 (weak), and a total washing time of approximately 45 min for a single wash. During the washing test, the samples were placed in a laundry net and washed separately. The detergent used was a neutral detergent (Wool Shampoo Original, AK, Republic of Korea) and its detailed composition is outlined in Table 1. Following the washing process, the samples were air-dried at room temperature. The washing cycles were repeated five times, and the surface resistance and dimensional changes were measured after each cycle. The dimensional changes of the fabrics due to the washing cycles were calculated as the ratio of the area of the samples after washing to the area of the original samples.

**Table 1:** Composition of detergent for washing durability test.

	Substances
Key substances	Water, Ethoxylated C12–14 alcohols, Sulphates, Sodiums (negative ions)
Preservatives	Sodium benzoate
Surfactant	Sodium alkylbenzene sulfonate (negative ion), Alkanole (C = 10–16) alkoxylated (C = 1–5) alkoxylated (C = 3–7) sulfate sodium (negative ion), Alcohol C12–14 ethoxylate (non-ion), Ethoxylated C10–16 alcohols sulphates sodium (negative ions), Myristic acid (negative ion)

## Results and discussion

### Appearance of polypyrrole-wool composites after various LBL cycles

**Figure 2:**
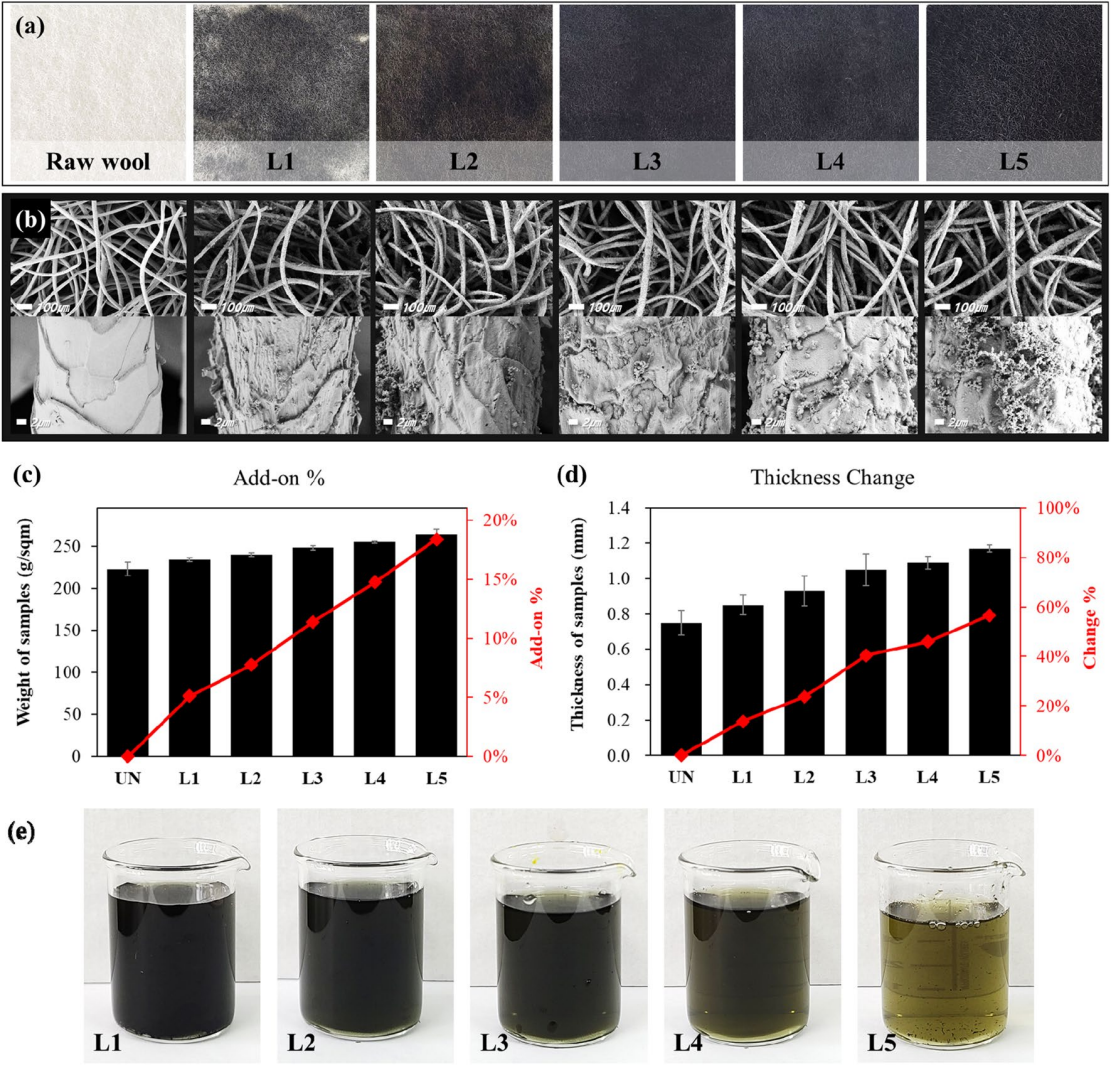
Appearance and mechanical properties of polypyrrole-wool composites samples after various LBL cycles; (**a**) photos, (**b**) FE-SEM images, (**c**) add-on, (**d**) thickness change, and (**e**) appearance of reaction solution used during pyrrole polymerization.

Figure 2a illustrates wool-felt samples after various LBL cycles. It can be confirmed that the cream-colored wool felt gradually turned black as the number of LBL cycles increased. The darker shades of the treated fabrics indicate an increased deposition of polypyrrole particles on the fabric as the number of LBL cycles increased. The non-uniform deposition of polypyrrole observed in the L1 and L2 samples was due to the presence of a hydrophobic epicuticle layer over each cuticle cell of the wool fiber^25^. The hydrophobicity of the epicuticular cell walls restricted the diffusion of water-dispersed pyrrole monomers into the fabric. As a result, while the pyrrole monomer was not uniformly absorbed onto the surface of the wool-felt, the polypyrrole particles formed by polymerization were unevenly distributed and stained. However, with additional LBL cycles, the hydrophilic polypyrrole promoted the absorption of the pyrrole monomer into the wool fiber, and more polypyrrole particles were deposited on the wool-felt^28^. Consequently, the overall color of the L3 sample was uniformly black. Thereafter, the color change was less noticeable.

The surface morphologies of the raw wool-felt and polypyrrole-wool composites after the LBL process were characterized by FE-SEM, as shown in Fig. 2b. The surface of the raw wool felt exhibited a scaly structure with a relatively smooth and clear surface. Because felt is composed of scaled wool fibers and forms a network structure, it can provide an abundant surface area for the deposition of polypyrrole^13^. After polymerization, the deposited polypyrrole formed a continuous layer with irregular granules on the wool fibers via in-situ polymerization. Generally, polypyrrole formed granular particles located along the fiber surface or in the pores between the fibers. As the number of LBL cycles increased, more polypyrrole particles were attached to the wool fiber, ultimately forming more polypyrrole particle agglomerates. As a result, the add-on % and the thickness of the polypyrrole-wool composites increased along with the number of LBL cycles, as shown in Fig. 2c,d.

Wool is composed of a single protein, keratin, which is a copolymer of 18 amino acids: glycine, alanine, serine, proline, cystine, and tyrosine; therefore, wool fibers offer abundant active sites to form chemical interactions with polypyrrole^17,29,30^. Hydrogen bonds were presumably formed between the lone pairs of electrons on the various N and O atoms in the pyrrole rings of the polypyrrole molecules and amino acids of the wool protein^30–32^. These intermolecular bonds establish a durable deposition between the fibers and polypyrrole that easily enable the attachment of additional polypyrrole to the fiber as the number of LBL cycles increases. As shown in Fig. 2e, the reaction solution gradually became clearer as the number of LBL cycles increased, because the remaining polypyrrole that could not be deposited on the wool-felt decreased. It has been reported that the in-situ polymerization comprises two competitive processes: (1) the physical adsorption of monomers and polymers onto the fibers and (2) the polymerization in the solution at the initial stage of the polymerization reaction^32^. That is, polymerization on the fiber increased more than polymerization in the solution as the number of LBL cycles increased. In the early stages of polymerization, polypyrrole can easily access the functional groups of wool fibers due to the porous and bulky structure of felt^30^. Subsequently, as the number of LBL cycles increased, the opportunity for direct interacting between the fibers and polypyrrole decreased because of the constantly decreasing surface area of the wool fibers. On the other hand, the bonding strength between the polypyrrole already deposited on the wool fibers and the additionally generated polypyrrole increased, and the add-on % and thickness of the composites increased linearly. As a result, the introduction of the LBL method for the development of polypyrrole-wool composites has enabled the uniform and even deposition of a substantial amount of polypyrrole onto the surface of wool fibers.

### Chemical compositions

The chemical composition and surface changes of the wool-felt fabrics after the deposition of polypyrrole were examined by FT-IR and EDX analyses.

**Figure 3:**
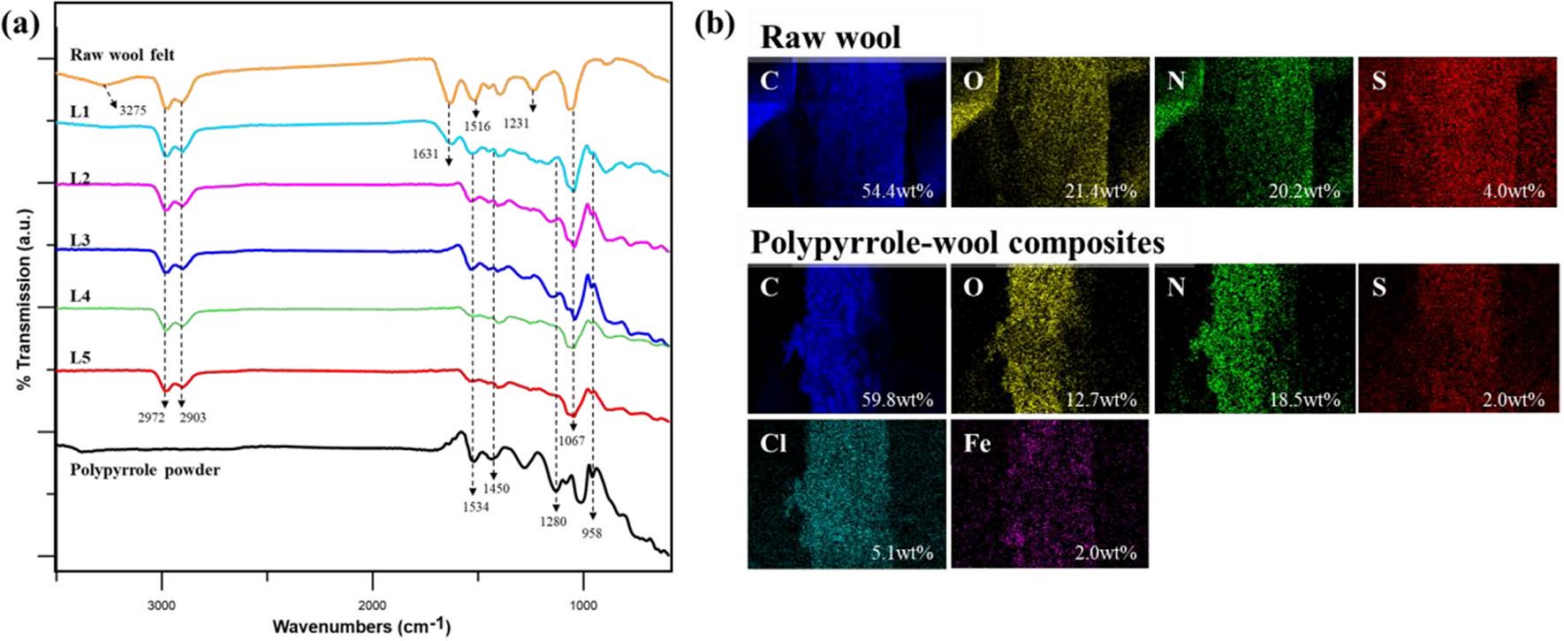
(**a**) FT-IR spectra and (**b**) EDX analysis results of polypyrrole powder, raw wool felt, and polypyrrole-wool composites according to the number of LBL cycles.

The FT-IR spectra of raw wool felt, polypyrrole powder, and polypyrrole-deposited wool fabrics are shown in Fig. 3a. The FT-IR spectra of the raw wool felt showed typical features of keratin. Amide A gave spectral bands at 3275 cm^−1^, assigned to the combined stretching vibrations of N–H and O–H bonds, which fall under hydrogen bond formation^33,34^. The peaks at 2972 cm^−1^ and 2903 cm^−1^ represent the Amide B (C–H)^35^. Peptide (–CONH–) groups were observed at 1631 cm^−1^, 1516 cm^−1^, and 1231 cm^−1^, assigned to I, II, and III amides, respectively^36,37^. The peak at 1067 cm^−1^ corresponds to the S–O vibrations^37^. Meanwhile, the spectra of all polypyrrole-wool composites spectra due to Amide A, which were present in the raw wool-felt, disappeared^24^. New bands appeared at 1534, 1450, and 1280 cm^−1^, attributable to the C=C ring stretching, C–C stretching, and C–N stretching of polypyrrole^36,38^. Therefore, the characteristic peaks of polypyrrole were observed in the FT-IR spectra of the polypyrrole-wool composites, confirming that polypyrrole was properly polymerized on the wool-felt surface.

Figure 3b shows the results of the EDX analysis that was conducted to characterize the surface element distribution of the raw wool felt and polypyrrole-wool composites. Compared to the raw wool felt, the carbon of the polypyrrole-wool composites was increased, whereas their oxygen and nitrogen decreased^13^. Sulfur, which is naturally present in wool keratin, also decreased with the deposition of polypyrrole^36^. In addition, chlorine was embedded in the polypyrrole molecules in the form of dopant anions^39^. Furthermore, a small amount of iron was introduced in the oxidation of pyrrole monomer by FeCl_3_·6H_2_O^13,36^. Quantitative analysis and elemental mapping confirmed that polypyrrole was well-polymerized and evenly distributed on the wool fibers surface.

### Mechanical properties

The changes in the mechanical properties of the wool-felt according to the polypyrrole deposition and the number of the LBL cycles were confirmed by tensile strength and bending rigidity tests.

**Figure 4:**
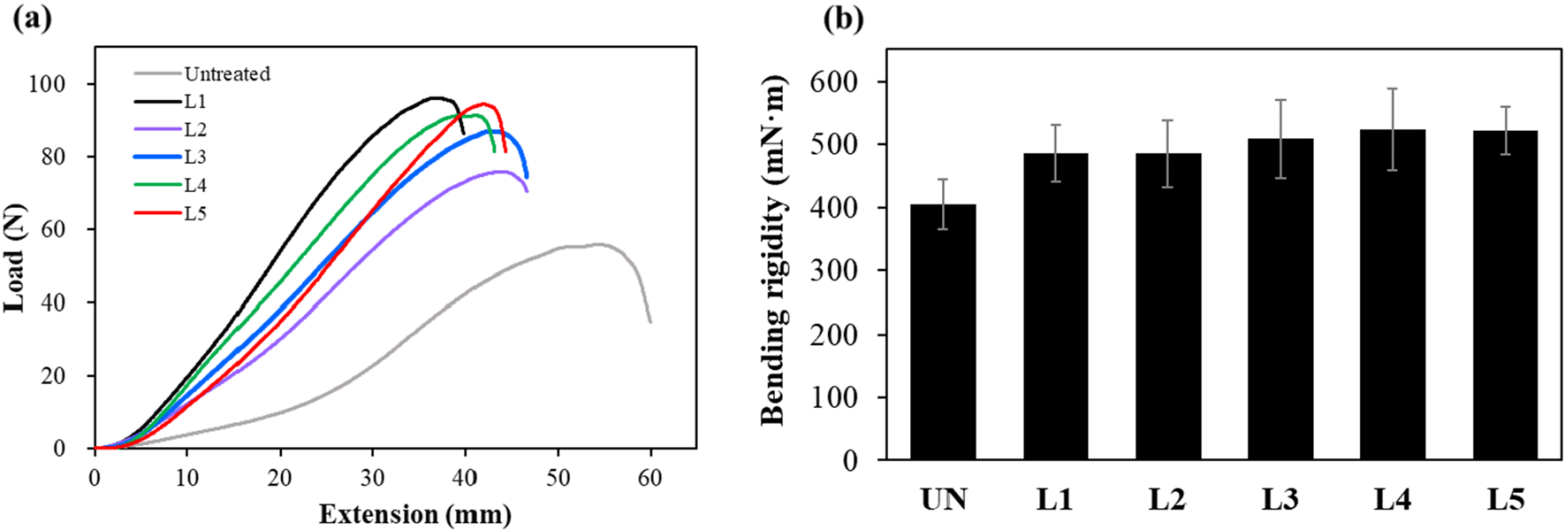
(**a**) Tensile strength and strain curves and (**b**) bending rigidity of the samples according to the number of LBL cycles.

Figure 4a shows the tensile strength and strain curves of each sample with respect to the number of LBL cycles. Generally, wool fibers are moderately strong because of their low tensile strength and good elasticity^40^. Here, the raw wool fabrics started with an excellent strain value of approximately 45%; however, as polypyrrole was continually deposited on the wool fabrics, the tensile strength increased and the strain decreased for all samples, regardless of the number of LBL cycles. Conductive polymers such as polyaniline and polypyrrole can be used to enhance the tensile strength of natural fibers via chemical oxidative deposition; particularly, polypyrrole coatings have been reported to improve the tensile strength of the wool fibers^8,30^. This is because polypyrrole is rigid substance with a conjugated double-bonded structure^11^. Because free-standing films of polypyrrole have high tensile strength, the increase in the toughness of polypyrrole-coated wool fabric is attributed to the reinforcing effect of the high-strength and high-modulus conductive polymer coating on the fiber^40^. Therefore, it is considered that the polypyrrole particles formed through in-situ polymerization disperse inside the fabric to form a rigid conductive layer and stiffen the fabric by restricting the movement of yarns. In particular, the wool-felt were nonwoven fabrics formed by the entanglement of fibers with a random orientation and a three-dimensional structure^41^. Therefore, when the polypyrrole particles were deposited in the pores formed between the fibers, a continuous polypyrrole layer was better formed inside the fabric, making the wool felt more rigid. As a result, the polypyrrole-wool composites better resisted external forces, but their elasticity was reduced. Meanwhile, the clear correlation between the number of LBL cycles and the changes in strength and strain has not been firmly established. Wool is prone to damage and a reduction in strength due to oxidants such as FeCl_3_^30^. Consequently, the repeated immersion in oxidant solutions following the LBL cycles may reduce the strength of wool felt. However, in this study, as LBL cycles were repeated, the positive impact on strength enhancement through the formation of polypyrrole layer was counteracted by a concurrent decrease in elasticity due to fiber damage caused by oxidation. As a result, there was no distinct variation in strength and strain depending on the number of LBL cycles.

This tendency was also observed in the bending rigidity evaluation results, as shown in Fig. 4b Compared with the untreated raw wool felt, the bending rigidity of all the polypyrrole-wool composites was increased by 20% or more. The bending stiffness of a fabric is affected by various factors such as the initial modulus, composition, weight, and thickness^11^. As mentioned above, the initial modulus, tensile strength, weight, and thickness of the polypyrrole-wool composites increased owing to the deposition of rigid polypyrrole, resulting in increased bending rigidity and decreased flexibility.

During the first LBL deposition cycle (L1), the strength increased and the strain decreased rapidly; however, the strength decreased and the strain increased after the second deposition cycle (L2). Thereafter, as the LBL cycles progressed, the strength increased and the strain decreased. The changes observed during L1 clearly originated from polypyrrole deposition. The changes during L2 caused mechanical damage to the wool-felt, stemming from the oxidizing agent’s action during the polymerization of polypyrrole, which was not compensated for by the improved mechanical properties due to the increased deposition of polypyrrole. Because the ferric chloride used during polymerization is a strong oxidant, it can cause the oxidative decay of wool^42^. However, as the polypyrrole penetrated not only the surface of the fabric but also its interior throughout the LBL cycles, the amount of polypyrrole deposited on the fabric increased, and as the thickness of the polypyrrole-wool composites also increased, causing the tensile strength to gradually increased again. Regarding the bending rigidity, the stiffness showed a gradual increase with the number of LBL cycles, although the rate was insignificant. Nevertheless, it is determined through this study that the introduced LBL method did not significantly alter the mechanical properties of the polypyrrole-wool composites.

### Surface resistivity

Wool-felt behaves as an insulator with infinite resistance. However, when polypyrrole is deposited on the surface of the wool fabric through in-situ polymerization, current flows owing to the π-conjugated structure of polypyrrole^11^. As a result, the surface resistance of the polypyrrole-wool composites was significantly reduced after the deposition of polypyrrole, as shown in Table 2. In addition, as the number of LBL cycles increased, the surface resistance of the polypyrrole-wool composites gradually decreased.

**Table 2:** Surface resistance of the samples according to the number of LBL cycles.

	Raw wool	Polypyrrole-wool composites				
		L1	L2	L3	L4	L5
Surface resistance (Ω/sqm)	∞	442 ± 71	153 ± 18	74 ± 9	72 ± 11	73 ± 3
Reduction of change (%)	–	100%	35%	17%	16%	17%

**Figure 5:**
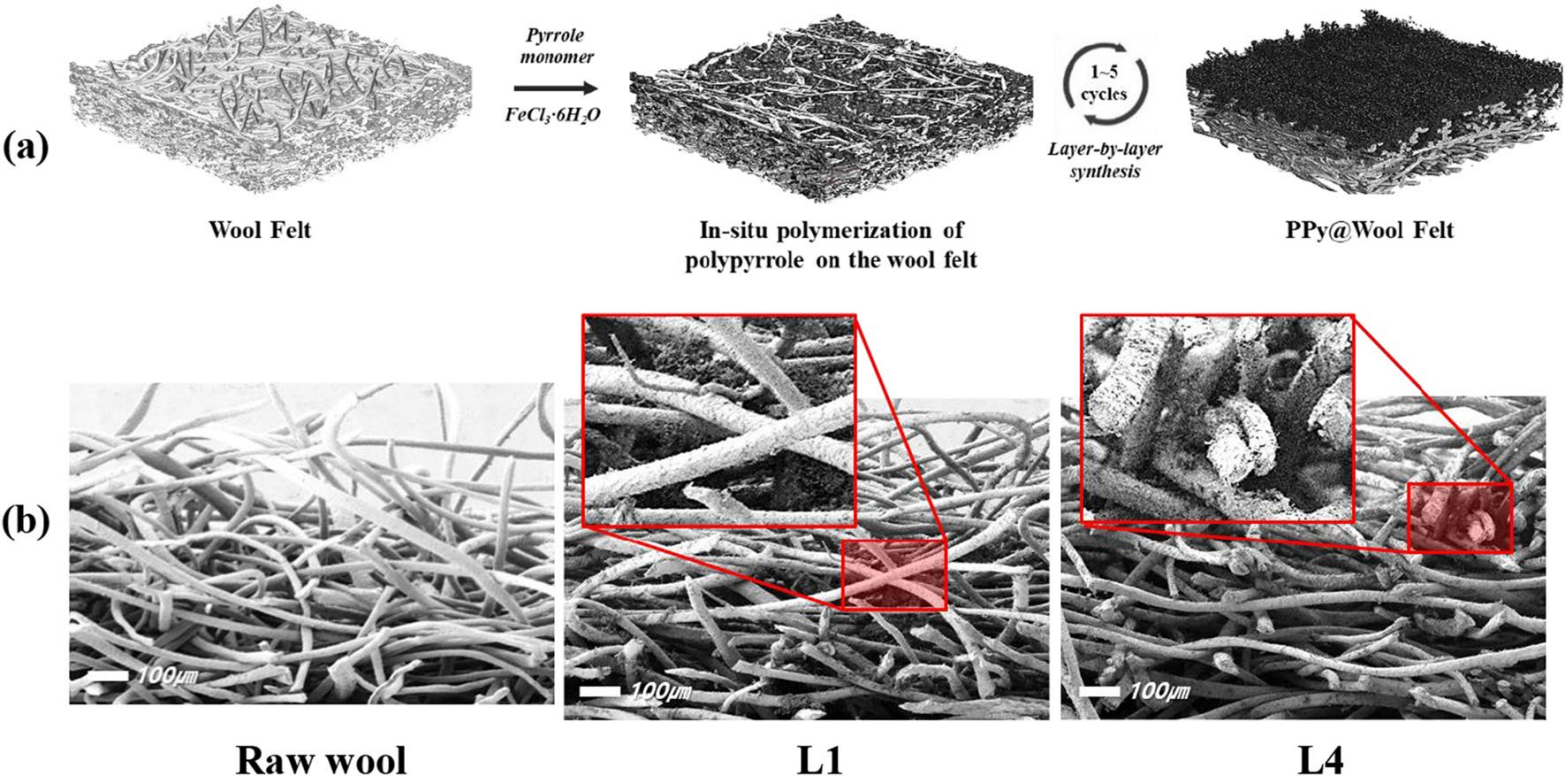
(**a**) Schematic of the polypyrrole polymerization process on the wool-felt and (**b**) FE-SEM images of the cross-section of the samples.

Polypyrrole has a conjugated structure with alternating single and double bonds^43^. The π electrons in the conjugated double bond are not fixed to a specific carbon atom, and as such they can be transferred from one carbon–carbon bond to another, meaning that they tend to extend across the entire molecular chain^14^. Therefore, the longer the chain of continuously connected polypyrrole molecules, the smoother the flow of electrons, resulting in improved conductivity. During the L1 cycle, a relatively high surface resistance value was obtained as the current flowed. This was attributed to the non-uniform deposition of polypyrrole, that impeded the formation of a continuous conductive layer on the wool-felt. Unevenness is a key issue in the coating of wool fibers with conductive polymers, owing to their hydrophobic nature^25^. However, the non-uniformity of the coating improved as the LBL cycle was repeated, resulting in a gradual improvement in conductivity. This was because polypyrrole was uniformly and abundantly deposited on the surface and pores of the fabric as the LBL cycles progressed, thus effectively forming a conductive layer. Figure 5 illustrates cross-sectional SEM images of the polypyrrole-wool composites according to the number of LBL cycles. Wool-felt has a three-dimensional structure in which fibers and pores are randomly distributed, owing to the entanglement of the fibers^41^. When polypyrrole was deposited on the wool-felt, it initially moved through the pores, as they were easier to penetrate than the fiber surface. The repeated LBL cycles caused the abundant polypyrrole to polymerize and progressively fill the pores of the wool felt, until it started to deposit on the fiber surfaces. These results indicate that the conductivity of the polypyrrole-deposited composites was affected by the polypyrrole deposited on the fabric surface and the polypyrrole impregnated into the fabric, indicating that the continuity and connectivity of the conductive particles deposited on the surface and inside of the fabric are important elements for improving the conductivity of polypyrrole composites^11^. In particular, the rapid decrease in surface resistance up to L3 was due to the sufficient surface area provided by the overhead structure of the wool felt and the increased contact points in the polypyrrole network distributed on the fiber surface and pores^13^. However, L4 and L5 showed similar surface resistances to L3, despite the larger add-on % that stemmed from the increased deposition amount of polypyrrole. After polypyrrole formed a conductive layer within the wool-felt to secure a sufficient path for electron movement, even if the amount of polypyrrole increased with additional polymerization, the change in conductivity was insignificant because the flow path of electrons remained the same^44^.

### Electric heating performance

The electrical heating performance of polypyrrole-wool composites is based on resistance heating via the Joule effect^2,10,11^. Resistance heating occurs as electrons move in a conductor under an electric field and collide with atomic nuclei, increasing their kinetic and vibration energies of atomic nuclei and releasing them as heat^45^. In particular, when polypyrrole is isotropically dispersed in a polymer matrix such as a fabric, the flow of free electrons generates heat on the surface of the fabric while generating chaotic heat motion without direction^11^.

**Figure 6:**
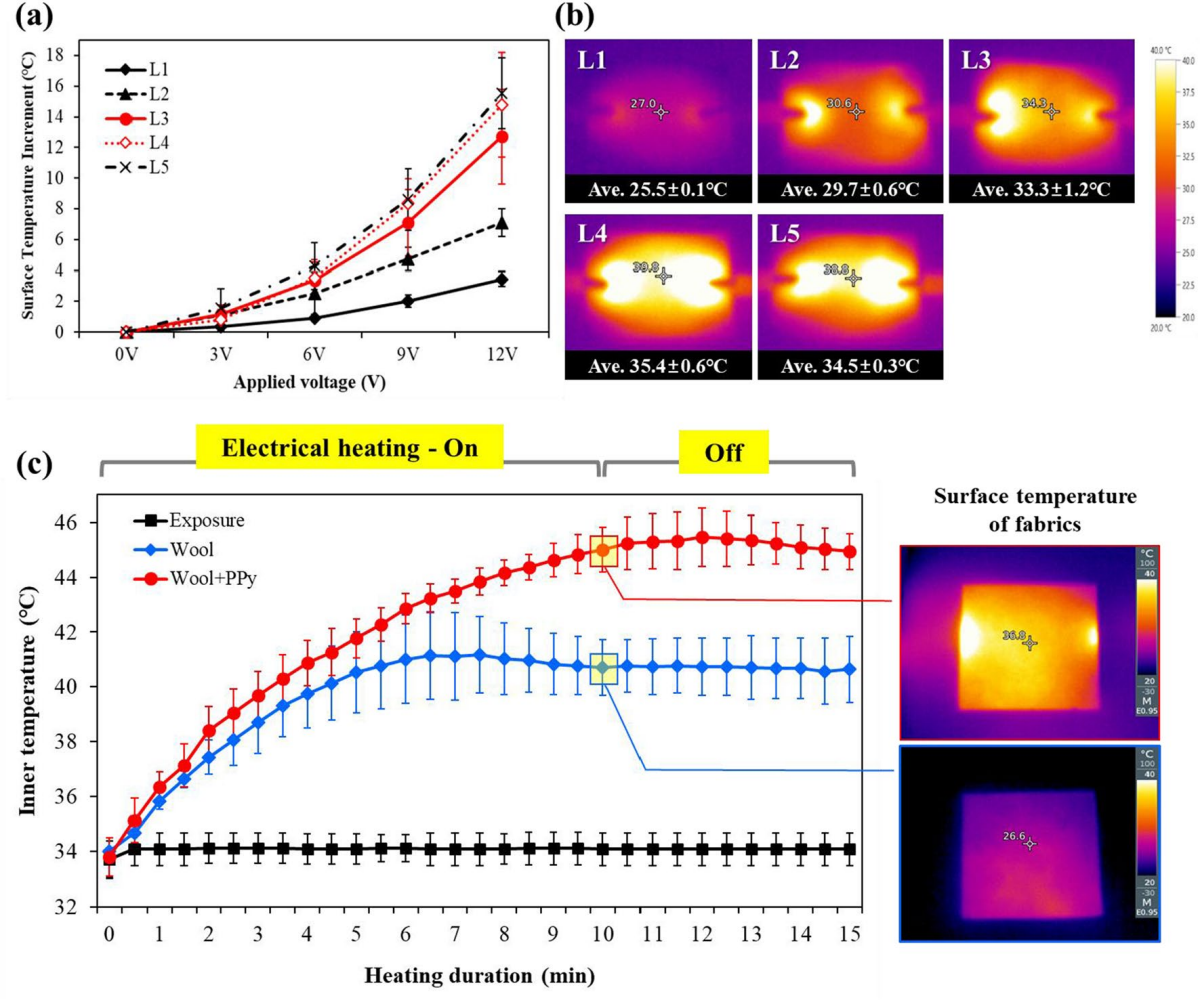
Electrical heating performance of polypyrrole-wool composites according to the number of LBL cycles; (**a**) surface temperature increment according to the applied voltage, (**b**) infrared thermal images and average surface temperature (applied voltage: 12 V), and (**c**) temperature change curve of the constant air layer according to sample treatment and electrical heating performance.

To evaluate the electrical heating performance according to the number of LBL cycles, voltage was applied to each sample, and the surface temperature was observed through infrared thermal images for 1 min. As shown in Fig. 6a, the surface temperature increases linearly with the applied voltage, regardless of the number of LBL cycles. According to the Joule effect, the amount of current applied to the sample increases as the voltage increases; therefore, the amount of heat generated also increases^46,47^. When 3 V was applied, the surface temperature increment did not show a significant difference according to the number of LBL cycles, but the difference became clear as the applied voltage increased. When 12 V was applied, the more LBL cycles, the higher the surface temperature, indicating excellent electrical heating performance. As shown in Fig. 6b, when a voltage was applied after equilibrating with the environment at a temperature of 21.9 ℃, the average surface temperature of each sample was 25.5 ℃ for L1, 29.7 ℃ for L2, 33.3 ℃ for L3, 35.4 ℃ for L4, and 34.5 ℃ for L5, which was 3.4 ℃, 7.1 ℃, 12.7 ℃, 14.8 ℃, and 15.5 ℃ higher than its initial temperature, respectively. Increasing the electric field above a certain voltage, the so-called hot voltage, depends on the amount of polypyrrole in the fabrics^12^. That is, a higher polypyrrole content in the wool-felt results in an increase in the Joule effect and consequently increases the surface temperature. Therefore, the greater the polypyrrole, the lower the surface resistance, the greater the electron flow, and better the resistance heating effect by collision with the atomic nucleus.

However, L4 and L5 showed similar electroheating behaviors despite the different amounts of deposited polypyrrole. These trends are consistent with the surface resistance results. As the increased polypyrrole within the wool felt at L4 sufficiently secured a current path, even if the amount of polypyrrole increased at L5, it did not affect the conductivity improvement^44^. Therefore, after L4, only changes in mechanical properties, such as weight increase and strain decrease, occur without improvement in conductivity and heating performance; therefore, L4 was selected as the optimal condition for electrical heating of the polypyrrole-wool composites. L4 showed a maximum surface temperature of 41 ℃ even at a relatively low voltage of 12 V, and the average temperature increment was 15 ℃ compared to before voltage application. In addition, a uniform temperature distribution was observed as listed in Fig. 6b. Because the safe voltage of the human body is 36 V, polypyrrole-wool composites are very safe and are expected to serve as a smart clothing or wearable devices that warm the human body in a cold environment^47^. In addition, the desired heat-generation temperature can be controlled by varying the amount of polypyrrole deposited on the wool felt by adjusting the number of LBL cycles or applied voltage.

The electrical heating performance of the polypyrrole-wool composites can be applied to smart clothing by warming the human body in a cold environment. In order to investigate the change in temperature inside the clothing according to the electrical heating performance of polypyrrole-wool composites, the hot plate was set at 35 ℃, which is the skin temperature, covered with raw wool-felt or L4, and the temperature of the air between the hot plate and the fabric was measured, respectively.

As shown in Fig. 6c, the temperature of the air layer on the exposed hot plate surface was kept constant at 34 ℃. On the other hand, when covered with raw wool felt, the temperature of the air layer gradually increased until the first 7 min, and reached equilibrium at about 40 ℃. Because the environmental temperature is lower than the human skin temperature, heat moves from the skin to the environment, resulting in heat loss. If an insulator such as a fabric is placed between the skin and the environment, heat cannot move and is absorbed and reflected by the insulator, thereby increasing the inner temperature and exhibiting warmth^46^. Wool fibers have a low thermal conductivity because they lack the ability to conduct effective thermal energy owing to the localization of electrons in their chemical structures^48^. In this study, the effect of increasing the inner temperature by about 6 ℃ was observed even with the raw wool-felt alone, confirming the excellent insulation performance.

This wool insulation effect satisfied the electrical heating properties of polypyrrole and demonstrated a much more effective heating performance. When the polypyrrole-wool composites (L4) was covered and voltage was applied, the average surface temperature was about 39 ℃ due to electric heating, and the temperature of the air layer rapidly increased to a maximum of 46.6 ℃. In addition, this heating effect continued even after application of the voltage was terminated, maintaining the internal temperature of about 45 ℃. The polypyrrole deposition enhances the thermal conductivity of wool fabrics^30,49^. In previous studies, the typical thermal conductivity value of uncoated wool fabric was 0.0312 W/m K, whereas the wool fabric after polypyrrole coating was about 1.6 W/m K, 51 times increase over the control fabric^31^. Therefore, the polypyrrole-deposited wool felt could better transmit the heat from the hot plate and the heat generated by resistance heating to the inside of the fabric. Consequently, as shown in the thermal image in Fig. 6c, the entire surface of the fabric heated quickly and more evenly. Insulating materials play a significant role in the design of conductive fabrics to reduce heat loss to the surroundings and enable prolonged use with low power inputs in practical applications^49^.

### Evaluation of conductivity preservation

**Figure 7:**
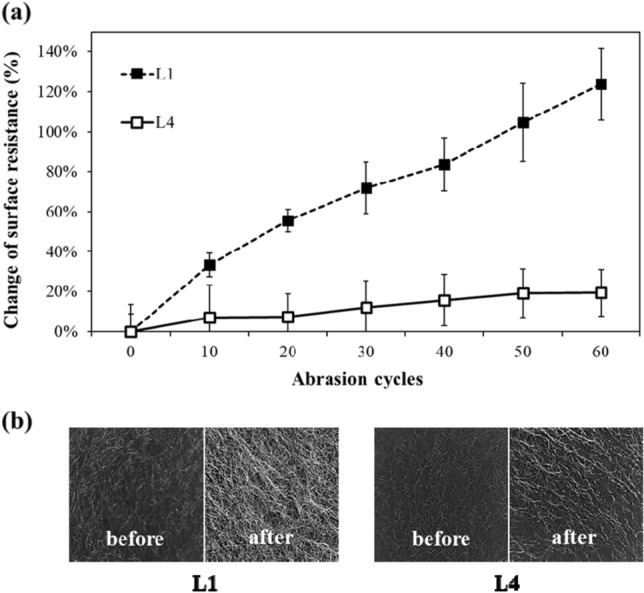
(**a**) Change of surface resistance and (**b**) surface images of polypyrrole-wool composites according to abrasion cycles.

The polypyrrole molecules deposited on the fabric can detach from the surface owing to external forces encountered in daily use, such as friction, rubbing, scratching, and compression^46^. Because the conductivity may deteriorate during this process, it is necessary to confirm the degradation caused by repetitive external forces. To evaluate the durability of the conductive fabrics with use and maintenance, polypyrrole-wool composites samples were subjected to abrasion tests using a grinder. Figure 7 illustrates the surface resistance and appearances as a function of abrasion for L1 and L4, selected under optimal conditions, to verify the effect of LBL method.

In the case of L1, the surface resistance increased linearly with the number of abrasion cycles and exhibited the greatest change. The average resistivity value of L1 increased by 124% after 60 abrasion cycles, from to 440 ± 52 to 984 ± 235 Ω/sqm. Through the visual appearance of abraded L1 in Fig. 7b, the polypyrrole on the fabric surface fell off significantly because of abrasion, and the cream-colored wool fiber was exposed, making the surface very rough. However, as the number of LBL cycles increased, the increase in surface resistance due to abrasion decreased. In the case of L4, the surface resistance only increased by about 20% from 72 ± 16 to 86 ± 33 Ω/sqm after 60 abrasion cycles. Such excellent conductivity durability can be guaranteed because the polypyrrole layer effectively grows around the individual fibers, even in the innermost part of the yarn, by gradually depositing polypyrrole from the surface to the inside of the wool-felt fabric through LBL cycles^50^. The polypyrrole conductive layer continuously formed up to the inside of the 3D felt structure was maintained even though the polypyrrole particles were eliminated owing to the abrasion of the surface; however, the increment of the surface resistance was relatively low.

**Figure 8:**
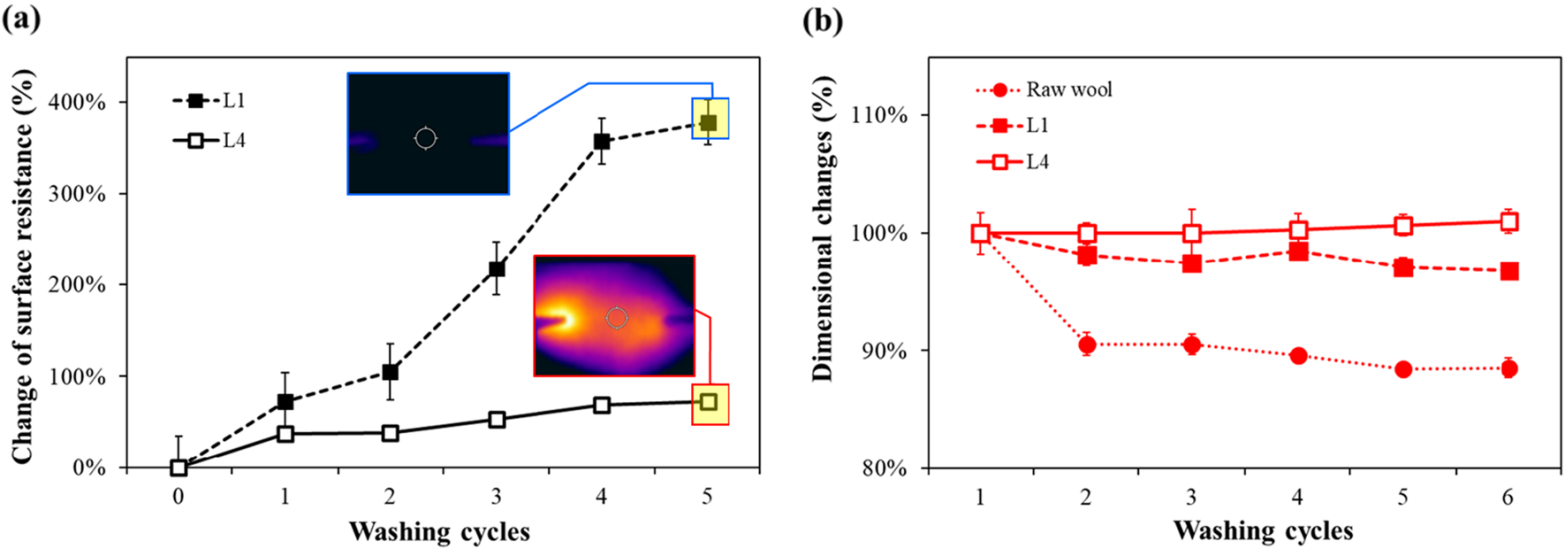
(**a**) Change of surface resistance and (**b**) dimensional change of polypyrrole-wool composites according to washing cycles.

As a textile material, washing is an essential maintenance process. In particular, wool fibers, with their scale structure on the fiber surface, may experience shrinkage due to mechanical forces and detergents during the washing process. Therefore, the polypyrrole-wool composites developed through this study should also maintain its performance and dimensional stability even through repetitive washing. Figure 8 illustrates the surface resistance and dimensional changes of L1 and L4 after 5 repeated washing cycles.

As depicted in Fig. 8a, the polypyrrole-wool composites exhibited a gradual increase in surface resistance due to repetitive washing. L1 and L4 demonstrated increments of 378% and 72%, resulting in values of 1912 ± 168 Ω/sqm and 112 ± 2 Ω/sqm compared to the pre-washing conditions, respectively. The observed loss of conductivity is attributed to the de-doping of polyprrole molecules by water and detergent, leading to a shortened conjugation structure^51,52^. Additionally, during the washing process, mechanical forces and fabric friction caused the detachment of polypyrrole particles from the fibers^53,54^. Zaman et al.^55^ categorize a washing cycle into four detrimental actions, highlighting potential interactions and interdependencies: mechanical stress, thermal stress, water stress, and chemical influence. Therefore, the complex interactions occurring among water, detergent, and wool fabric during the washing process influenced surface resistance. This influence was particularly pronounced in L1, where polypyrrole deposition appeared more uneven and less abundant. For L1, even after just one washing cycle, the surface resistance increased by over 73%, and thereafter, it continued to rise sharply. Consequently, after five washing cycles, the heating performance was lost. In contrast, L4 also experience an increase in surface resistance with repetitive washing; however, the change was not significant. Even after five washing cycles, the heating performance was maintained as depicted infrared thermal images in Fig. 8a. This can be attributed to the gradual and uniform formation of a more rigid polypyrrole layer on the wool felt surface and within, achieved through the LBL method.

However, even with a rigidly formed polypyrrole layer, it was inevitable to avoid chemical reactions induced by the detergent. The detergent used in this study contains various components, including not only surfactants but also acidity regulators and bleaching agents, aimed at enhancing washing efficiency^56^. Additionally, the reduction in the surface energy of the water due to the detergent facilitates easier penetration into the fabric, enabling more active chemical interactions. Therefore, considering the variation in surface resistance due to the chemical effects of the detergent, beyond mere friction, it is evident that further research is needed to explore additional management approaches for improvement.

It is noteworthy that the deposition of polypyrrole resulted in a reduction in the shrinkage of the wool felt during washing, maintaining its dimensional stability. As seen in Fig. 8b, raw wool experienced approximately 10% contraction due to friction and the action of the detergent during washing. In contrast, the shrinkage rate of the wool-polypyrrole composites was minimal, ranging from 1 to 3%, and in the case of L4, no observable changes in dimension were noted. This is because polypyrrole particles deposited on the wool fiber surface, covering the scale structure of the wool. As a result, the directional friction effect of the scales was lost, preventing the significant shrinkage^57^. Therefore, the dimensional changes in wool felt due to washing were somewhat improved by the deposition of polypyrrole. It is expected that his improvement will enable convenient maintenance when applied to smart clothing.

Through these tests, it is expected that the polypyrrole-wool composites made by conducting LBL cycles can be used as practical e-textile materials by confirming their excellent conductivity and durability, despite repeated surface abrasion and machine washing.

## Conclusions

In this study, the primary objective was to enhance the electrical heating and maintenance performance of conductive polymer-textile composites by leveraging the in-situ polymerization of polypyrrole within wool fabric, which inherently possesses superior thermal insulation properties. The LBL method was employed to achieve a uniform deposition of polypyrrole onto the surface of wool-felt fabric. Through systematic evaluation, the resulting polypyrrole-wool composites were scrutinized based on the number of LBL cycles, focusing on changes in appearance, mechanical and chemical properties, and conductivity, including surface resistance, heat performance, and conductivity preservation.

As a result of observing the appearance, add-on %, and thickness change of the polypyrrole-wool composites, initial polymerization cycles exhibited uneven distribution, but as the number of LBL cycles increased, a more uniform deposition occurred, with enhanced distribution on both the fiber surface and pores. Chemical composition analysis confirmed the successful engagement of polypyrrole through pyrrol polymerization, forming chemical interactions with wool molecules.

Significant mechanical property changes were observed in wool-felt, indicating increased tensile strength and decreased strain as a rigid conductive layer formed through polypyrrole deposition. Interestingly, this effect demonstrated a consistent level irrespective of the number of LBL cycles does not notably alter the mechanical properties of wool fabric. In addition, the bending rigidity increased and the flexibility decreased owing to the increase in the strength, weight, and thickness of the polypyrrole-wool composites. This effect strengthened as the number of LBL cycles increased.

The insulating properties of wool-felt were effectively countered by the deposition of polypyrrole, resulting in a significant decrease in surface resistance as current flowed through. Notably, the LBL method contributed to a continuous polypyrrole network formation, enhancing conductivity and positively impacting the conductivity preservation performance. Even after rigorous testing, the maintenance surface resistance exhibited only a 20% increase after 60 cycles of abrasion tests, and a 70% increase after five washing, indicating exceptional conductivity durability.

The surface temperature of the polypyrrole-wool composites increased due to resistance heating under current flow. Higher applied voltage and increased LBL cycles resulted in a superior heating effect, showcasing high surface temperatures and uniform distribution. This heating mechanism leveraged the matching electrical properties of polypyrrole with the insulation characteristics of wool, realizing a potent and uniform heating effect. Importantly, this study confirmed wool felt’s ability to efficiently transfer heat from the heating element and polypyrrole-generated heat to the fabric’s interior.

In conclusion, this study confirmed the efficacy of the LBL method in uniformly depositing polypyrrole on wool, capitalizing on the inherent advantages of wool fabric. The conductive polymer-textile composites developed hold great potential for applications in smart clothing, upholstery, medical products, and healthcare materials for thermal treatments, offering an optimal balance of electrical properties, flexibility, and durability.

## Data Availability

The datasets generated during and/or analysed during the current study are available from the corresponding author on reasonable request.

## References

[1] Erodogan MK, Karakisla M, Sacak M (2018). Polypyrrole and silver particles coated poly(ethylene terephthalate) nonwoven composite for electromagnetic interference shielding. J. Compos. Mater..

[2] He H, Guo Z (2023). Fabric-based superhydrophobic MXene@polypyrrole heater with superior dual-driving energy conversion. J. Colloid. Interface. Sci..

[3] Babu KF, Dhandapani P, Maruthanmuthu S, Kulandainathan MA (2012). One pot synthesis of polypyrrole silver nanocomposite on cotton fabrics for multifunctional property. Carbohydr. Polym..

[4] Shi J, Liu S, Zhang L, Yang B, Shu L, Yang Y, Ren M, Wang Y, Chen J, Chen W, Chai Y, Tao X (2020). Smart textile-integrated microelectronic systems for wearable applications. Adv. Mater..

[5] Siavashani VS, Nevin G, Montazer M, Altay P (2022). Highly stretchable conductive fabric using knitted cotton/lycra treated with polypyrrole/silver NPs composites post-treated with PEDOT:PSS. J. Ind. Text..

[6] Shi Q, Sun J, Hou C, Li Y, Zhang Q, Wang H (2019). Advanced functional fiber and smart textiles. Adv. Fiber. Mater..

[7] Grancaric AM, Jerkovic I, Koncar V, Cochrane C, Kelly FM, Soulat D, Legrand X (2018). Conductive polymer for smart textile applications. J. Ind. Text..

[8] Shah MA, Pirzada BM, Price G, Shibiru AL, Qurashi A (2022). Applications of nanotechnology in smart textile industry: A critical review. J. Adv. Res..

[9] Garg S, Hurren C, Kaynak A (2007). Improvement of adhesion of conductive polypyrrole coating on wool and polyester fabrics using atmospheric plasma treatment. Synth. Met..

[10] Xie J, Pan W, Guo Z, Jiao SS, Yang LP (2019). In situ polymerization of polypyrrole on cotton fabrics as flexible electrothermal materials. J. Eng. Fibers. Fabr..

[11] Lee S, Park CH (2019). Conductivity, superhydrophobicity and mechanical properties of cotton fabric treated with polypyrrole by in-situ polymerization using the binary oxidants ammonium peroxodisulfate and ferric chloride. Text. Res. J..

[12] Wang Y, Jiang H, Tao Y, Mei T, Liu Q, Liu K, Li M, Wang W, Wang D (2016). Polypyrrole/poly(vinyl alcohol-co-ethylene) nanofiber composites on polyethylene terephthalate substrate as flexible electric heating elements. Compos. A Appl..

[13] Wu Z, Zeng Y, Liu Y, Xiao H, Zhang T, Lu M (2021). Utilization of waste wool felt architecture to synthesize self-supporting electrode materials for efficient energy storage. New. J. Chem..

[14] Ji F, Guo X, Liu A, Xu P, Tan Y, Wang R, Hao L (2021). In-situ synthesis of polypyrrole/silver for fabricating alginate fabrics with high conductivity, UV resistance and hydrophobicity. Carbohydr. Polym..

[15] Omastova M, Mosnackova K, Fedorko P, Trchova M, Stejskal J (2013). Polypyrrole/silver composites prepared by single-step synthesis. Synth. Met..

[16] Maity S, Chatterjee A (2018). Conductive polymer-based electro-conductive textile composites for electromagnetic interference shielding: A review. J. Ind. Text..

[17] Bai R, Yu Y, Wang Q, Yuan J, Fan X (2018). Laccase-mediated in situ polymerization of pyrrole for simultaneous coloration and conductive of wool fabric. Text. Res. J..

[18] Bober P, Stejskal J, Sedenkova I, Trchova MM, Martinkova L, Marek J (2015). The deposition of globular polypyrrole and polypyrrole nanotube on cotton textile. Appl. Surf. Sci..

[19] Serrano-Claumarchirant JF, Munoz-Espi R, Cantarero A, Culebras M, Gomez CM (2023). Electrochemical deposition of conductive polymers on fabrics. Coating.

[20] Wang Y, Peng HK, Li TT, Shiu BC, Zhang X, Lou CW, Lin JH (2020). Layer-by-layer assembly of low-temperature in-situ polymerized pyrrole coated nanofiber membrane for high-efficiency electromagnetic interference shielding. J. Porg. Coat..

[21] He J, Li R, Gu F (2013). Preparation of polyaniline/nylon conducting fabric by layer-by-layer assembly method. J. Appl. Polym. Sci..

[22] Wang J, Kaynak A, Wang L, Liu X (2006). Thermal conductivity studies on wool fabrics with conductive coatings. J. Text. Inst..

[23] Pan Y, Wang W, Gong K, Hurren CJ, Li Q (2019). Ultrasonic scouring as a pretreatment of wool and its application in low-temperature dyeing. Text. Res. J..

[24] Chatterjee A, Maity S (2017). A comparative study of reaction kinetics of in-situ chemical polymerization of polypyrrole onto various textile fibres. Surf. Coat. Technol..

[25] Navik R, Shafiq F, Khan A, Datta M, Peng X, Kamruzzaman M, Cai Y (2017). Preparation and characterizaions of polypyrrole on liquid ammonia pre-treated wool fabric. Fibers. Polym..

[26] Ehsanimehr S, Sonnier R, Najafi P, Ducos F, Badawi M, Formela K, Saeb MR, Vahabi H (2022). Layer-by-layer polymer deposited fabrics with superior flame retardancy and electrical conductivity. React. Funct. Polym..

[27] Huang G, Liu L, Wang R, Zhang J, Sun X, Peng H (2016). Smart color-changing textile with high contrast based on a single-sided conductive fabric. J. Mater. Chem. C.

[28] Thombare JV, Rath MC, Han SH, Fulari VJ (2013). Synthesis of hydrophilic polypyrrole thin films by silar method. Mater. Phys. Mech..

[29] Allafi F, Hossain MS, Lalung J, Shaah M, Salehabadi A, Ahmad MI, Shadi A (2022). Advancement in applications of natural wool fiber: Review. J. Nat. Fibers..

[30] Maity S, Singha K, Pandit P, Thomas S, Jose S (2022). Conductive polymer-coated wool composites for novel applications. Wool Fiber Reinforced Polymer Composites.

[31] Johnston JH, Kelly FM, Moraes J, Borrmann T, Flynn D (2006). Conducting polymer composites with cellulose and protein fibres. Curr. Appl. Phys..

[32] Lin T, Wang L, Wang X, Kaynak A (2005). Polymerising pyrrole on polyester textiles and controlling the conductivity through coating thickness. Thin Solid Films.

[33] Zhang Y, Zhang N, Wang Q, Yu Y, Wang P, Yuan J (2020). A facile and controllable approach for surface modification of wool by micro-dissolution. Fibers. Polym..

[34] Gashti MP, Ghehi ST, Arekhloo SV, Mirsmaeeli A, Kiumarsi A (2015). Electromagnetic shielding response of UV-induced polypyrrole/silver coated wool. Fibers. Polym..

[35] Lis MJ, Caruzi BB, Gil GA, Samulewski RB, Bail A, Scacchetti FAP, Moises MP, Bezerra FM (2019). In-situ direct synthesis of HKUST-1 in wool fabric for the improvement of antibacterial properties. Polymers.

[36] Varesano A, Aluigi Am Tonin C, Ferrero F (2006). FT-IR study of dopant-wool interactions during PPy deposition. Fibers. Polym..

[37] Motaghi Z, Eskandarnejad S, Montazer M, Ardjmand M, Moghadam MB (2012). Mechanical slenderizing of coarse wool fibre and determination of its characteristics with FTIR and Raman spectroscopy. J. Text. Inst..

[38] Chougule MA, Pawar SG, Godse PR, Mulik RN, Sen S, Patil VB (2011). Synthesis and characterization of polypyrrole(PPy) thin films. Soft Nanosci. Lett..

[39] Lu J, Zhang L, Xing C, Jia G, Lu Z, Tian Q, Zhang S, Lv J (2022). Polypyrrole and cotton fabric-based flexible micro-supercapacitors. J. Appl. Polym. Sci..

[40] Kaynak A, Wang L, Hurren C, Wang X (2002). Characterization of conductive polypyrrole coated wool yarns. Fibers. Polym..

[41] Silva P, Navarro M, Bessa J, Coelho A, Cunha F, Fangueiro R (2022). Influence of fibre diameter on the wool-based felt properties. Mater. Circ. Econ..

[42] Yaghoubidoust F, Wicaksono DHB, Chandren S, Nur H (2014). Effect of graphene oxide on the structural and electrochemical behavior of polypyrrole deposited on cotton fabric. J. Mol. Struct..

[43] Street GB, Lindsey SE, Nazzal AI, Wynne KJ (1985). The structure and mechanical properties of polypyrrole. Mol. Cryst. Liq. Cryst..

[44] Maity S, Chatterjee A (2018). Polypyrrole functionalized polyester needlepunched nonwoven fabrics for electro-magnetic interference shielding. Polym. Compos..

[45] Zhu L, Zhang L, Wu L, Sun Y, Bai Z, Xu J, Liang G, Xu W (2014). Conductive cotton fabrics for heat generation prepared by mist polymerization. Fibers Polym.

[46] Lee S, Park CH (2018). Electric heated cotton fabrics with durable conductivity and self-cleaning properties. RSC Adv..

[47] Hao D, Xu B, Cai Z (2018). Polypyrrole coated knitted fabric for fobust wearable sensor and heater. J. Mater. Sci. Mater. Electron..

[48] Maity S (2017). Optimizaion of processing parameters of in-situ polymerization of pyrrole on woolen textile to improve its thermal conductivity. Prog. Org. Coat..

[49] Kaynak A, Zolfagharian A, Featherby T, Bodaghi M, Mahmud MAP, Kouzani AZ (2021). Electrothermal modeling and analysis of polypyrrole-coated wearable E-textiles. Materials.

[50] Boschi A, Arosio C, Cucchi I, Bertini F, Catellani M, Freddi G (2008). Properties and performance of polypyrrole (PPy)-coated silk fibers. Fibers. Polym..

[51] Liu Y, Zhao X, Tuo X (2017). Preparation of polypyrrole coated cotton conductive fabrics. J. Text. Inst..

[52] Patil AJ, Deogaonkar SC (2012). Conductivity and atmospheric aging studies of polypyrrole-coated cotton fabrics. J. Appl. Polym. Sci..

[53] Zeng C, Wang H, Zhou H, Lin T (2015). Self-cleaning, superhydrophobic cotton fabrics with excellent washing durability, solvent resistance and chemical stability prepared from an SU-8 derived surface coating. RSC Adv..

[54] Park S, Kim H, Lee S (2023). Changes in characteristics of silver conductive fabrics owing to perspiration and washing. RSC Adv..

[55] Zaman, S. U., Tao, X., Cochrane, C., & Koncar, V. Market readiness of smart textile structures – reliability and washability. *IOP Conference Series: Materials Science and Engineering*, vol. 459 012071. (2018)

[56] Yun C, Park S, Park CH (2013). The effect of fabric movement on washing performance in a front-loading washer. Text. Res. J..

[57] Rippon J, Gupta BS (2008). Friction, felting and shrink-proofing of wool. Friction in textile materials.

